# Nutrition Education Improves Knowledge of Iron and Iron-Rich Food Intake Practices among Young Adolescents: A Nonrandomized Controlled Trial

**DOI:** 10.1155/2023/1804763

**Published:** 2023-03-27

**Authors:** Michael Akenteng Wiafe, Charles Apprey, Reginald Adjetey Annan

**Affiliations:** ^1^Department of Nutritional Sciences, School of Allied Health Sciences, University for Development Studies, P. O. Box TL, 1350 Tamale, Ghana; ^2^Department of Biochemistry and Biotechnology, Kwame Nkrumah University of Science and Technology, Private Mail Bag, Ghana

## Abstract

**Introduction:**

Nutrition education targeting adolescents' health has the potential to enhance their well-being into adulthood. This study assessed the impact of nutrition education on the knowledge of iron and iron-rich food intake practices of adolescents living in rural communities in Ghana.

**Method:**

An intervention study was conducted among 137 adolescents; 69 were assigned to the intervention group and 68 to the control group. Participants and guardians in the intervention group were involved in the nutrition education programme for six months. Participants in both groups completed sociodemographic, knowledge of iron, and iron-rich food intake practice questionnaires at pre- and postintervention. Data were analyzed by chi-square and *t*-tests.

**Results:**

At postintervention, the mean knowledge score (*p* < 0.05) in the intervention group and control group was 5.3 ± 1.7 and 3.9 ± 1.9, respectively. Interventions (76%) and controls (46%) had good knowledge status. The mean knowledge score of participants with good knowledge status in the intervention group was 6.1 ± 0.8 (*p* < 0.05), and the control group was 5.6 ± 0.7 (*p* < 0.05). Forty-two percent of participants in the interventions and 26% in the controls had good food intake status. Participants with good food intake status had mean food intake scores of 3.2 ± 0.4 (*p* < 0.05) and 3.8 ± 0.7 (*p* < 0.05) for the intervention and control groups, respectively. Both groups increased and had the same mean food intake score (1.5 ± 1.4, *p* > 0.05), however, relatively higher in the intervention group.

**Conclusion:**

Nutrition education improved the knowledge of iron and iron-rich food intake practices of participants in the intervention group compared to the control group. Nutrition education should be a critical component in the management and prevention of micronutrient deficiency in adolescents.

## 1. Introduction

Adolescents' health is critical due to growth spurt, lean body mass development, and onset of menarche. These biological and physiological changes demand an increase in the intake of nutrient-dense foods; however, poor nutrition knowledge by most adolescents instigate misguided dietary practices contributing to excessive intake of micronutrient-poor diet [1, 2]. Adolescents in different geographical regions have been shown to have inadequate intakes of micronutrients, such as iron, vitamin C, zinc, potassium, and calcium; thus, they are unable to meet the recommended dietary allowance [3–6].

Poor intake of micronutrient-rich foods leads to avoidable nutritional deficiencies such as zinc, calcium, vitamin C, folic acid, vitamin A, and particularly iron deficiency anaemia [7–11]. Iron deficiency anaemia among adolescents leads to low cognitive function, physical activity, and earnings, poor pregnancy outcomes, and increased infections, alcohol intake, and practice of risky sexual behaviors [12–14].

To prevent iron deficiency anaemia in adolescents, food preference factors such as knowledge about food, dieting, body image, level of maternal education, parental influence, and eating with family and peers have to be well managed [15–21]. Food intake practices among adolescents have strong linkages with socioeconomic status, food taste, flavour, food skills, and food availability [22–24]. Adolescents showed inadequate intake of fruits, vegetables, and animal sources of foods while increasing intake of saturated fat, sugar, and salt [4, 5, 25–27]. A study demonstrated that poor knowledge of nutrition by early adolescents affects dietary habits [28].

To address micronutrient deficiency in adolescents, nutrition education is emphasized to take the center stage [9]. Studies indicate that although adolescents acknowledge the relevance of food and nutrition knowledge, they, however, do not apply the knowledge to food practices [22, 28]. Contrary outcomes from scientific research have been reported in the relationship between nutrition education and food intake practices among adolescents. An inverse relationship between nutrition knowledge and food intake practices have been reported in cross-sectional studies from Kenya, Iran, and Ghana [29–31]. A positve relationship between nutrition knowledge and food intake practices has been documented in intervention studies from Malaysia, Iran and Brazil [32–34]. However, other studies have demonstrated inverse relationship between nutrition knowledge and food intake practices [29–31]. Systematic reviews suggest that the difference in the inverse or positive relationships is largely due to the approach and the multiple strategies used in administering the nutrition intervention such as changes in the food environment; involvement of parents, families, teachers, and dieticians; and effective application of theoretical models [35, 36].

Nutrition education and awareness have been recommended as a cost-effective intervention to reducing micronutrient deficiencies in adolescents [37]. A systematic review warrants further studies on nutrition education among adolescents living in sub-Saharan regions [38]. Improvement in nutrition knowledge level of adolescents tends to influence healthy dietary practices [39, 40]. Nutrition education contributed to behavior change and improved micronutrient and macronutrient food intake recommendations [41]. A systematic review showed that nutrition education improved dietary habits of college students in developed countries [42]. Adolescents living in urban and rural communities in Ghana showed nutrition knowledge deficit [25, 43]. A nutrition education intervention study in Ghana, among adolescents in an urban community, showed improvement in nutrition knowledge and behavior and not practices [44]. The effect of nutrition education on the knowledge of iron and food intake practices of early adolescents has not been explored much in most rural communities in Ghana. The study aimed at assessing the impact of nutrition education on knowledge of iron and iron-rich food intake practices among young adolescents in rural communities in Ghana.

## 2. Materials and Methods

### 2.1. Study Design, Setting, and Participants

**Figure 1:**
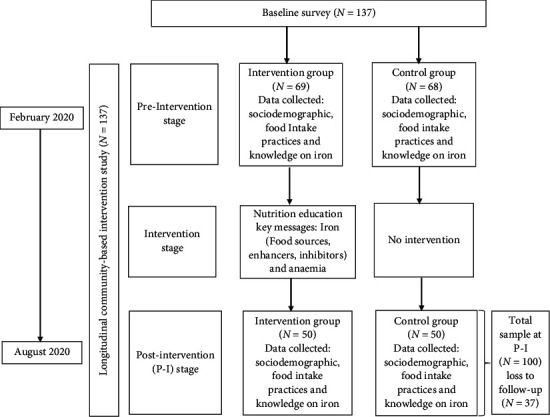
Overview of the study.

The study was a longitudinal community-based intervention study as summarized in Figure 1. The study targeted early adolescents (10-14 years old) living in Asante-Akim South Municipality in the Ashanti Region of Ghana. Juaso is the capital of the municipality. The municipality has about 51.4% of the population below the age of twenty. The municipality has one hundred and twenty communities and is predominantly agrarian [45].

### 2.2. Sampling Method

A power calculation showed that at least 84 participants were needed to detect statistically significant difference in knowledge level of iron between the intervention group (42 participants) and control group (42 participants). A total of 137 adolescents participated in the study after considering a dropout rate of 50%. A multistage sampling method was used to select participants from the communities and they were assigned to the control group (68) and intervention group (69) at a ratio of 1 : 1 using Microsoft Office Excel 2013.

### 2.3. Ethics

This study was conducted according to the guidelines laid down in the Declaration of Helsinki, and all procedures involving human participants/patients were approved by the Committee on Human Research Publications and Ethics of the Kwame Nkrumah University of Science and Technology, Ghana (reference number: CHRPE/AP/585/22). The aims and protocol of the study were explained to adolescents and their guardians. Assent of participants and consent of guardians were obtained verbally or through written documentation before being recruited into the study. Verbal consent was witnessed and formally recorded. The study was registered with the Pan African Clinical Trial Registry with a trial registration number PACTR202209659332435 and the URL https://pactr.samrc.ac.za/

### 2.4. Baseline Assessment

Trained researchers, dieticians, and nutritionists collected data on sociodemographic characteristics, knowledge of iron, and iron-rich food intake practices at baseline.

### 2.5. Data Collection

#### 2.5.1. Questionnaire

Sociodemographic characteristics, iron-rich food intake practices, and knowledge of iron data were collected by a structured questionnaire. The questionnaires about knowledge of iron and iron-rich food intake practices were adapted from a Food and Agriculture Organization (FAO) document titled “Guidelines for assessing nutrition-related knowledge, attitudes, and practices” [46].

#### 2.5.2. Dietary Data Collection

A 24-hour dietary recall questionnaire was used to collect data on iron-rich food intake practices.

#### 2.5.3. Iron-Rich Food Intake Practices

The iron-rich food intake practices were put under four main headings: organ meats, flesh meat, insects, and fish and seafood.

*(1) Scoring System*. Scores were used to evaluate the iron-rich food intake of participants. The total iron-rich food intake score was 4. Participants that had scores greater than or equal to three (≥3) were categorized as good iron-rich food intake status, and those that had scores less than or equal to two (≤2) were put in the poor iron-rich food intake status.

#### 2.5.4. Knowledge of Iron

Seven questions were asked about the knowledge of iron. The questions were in the form of multiple-choice and open-ended. It was put under four main categories: (1) iron deficiency anaemia (general signs and symptoms, causes, consequences, and prevention), (2) iron-rich foods, (3) iron-enhancing foods, and (4) iron-inhibiting foods.

*(1) Scoring System*. Scores were used to evaluate the knowledge level of iron of participants. The total knowledge score was 7. Participants having knowledge scores equal to or greater than five (≥5) were put in the good knowledge status, and participants having scores equal to or less than (≤ 4) were classified as having poor knowledge status.

### 2.6. Nutrition Education Intervention

Nutrition education programme was designed for participants and guardians in the intervention group. The intervention which was administered by dieticians and nutritionists lasted for six months. The nutrition education programme was structured as shown in Table 1. The purpose of nutrition education was explained to the participants and guardians. Nutrition education was delivered through group discussions, leaflets, charts, and posters. Meeting sessions per participant and guardian lasted for thirty to forty-five minutes. After the meeting, participants were given structured education materials. Participants were reviewed every three weeks, and a new topic was introduced for discussion as indicated in Table 1. The control group was also followed for six months. No intervention was administered to the control group.

### 2.7. Follow-Up and Postintervention Assessment

Postintervention data collection was done at six months of follow-up. Similar data collected at baseline were also collected at postintervention. Approximately 73% of participants showed up at postintervention, constituting 36.5% each of the controls [47] and interventions [47] (Figure 1).

### 2.8. Statistical Analysis

Data were analyzed using the Statistical Package for Social Sciences (SPSS version 25, Chicago, IL). A chi-square test was done to assess the relationship between sociodemographic characteristics, knowledge level of iron, and the study groups. An independent sample *t*-test was done to estimate the mean iron-rich food intake score and the knowledge status between the study groups and within the study groups. A paired *t*-test was conducted to estimate the mean knowledge score and mean food intake score within the study groups. Data are presented as mean, standard deviation, mean difference, frequency, and percentage. All *p* values less than 0.05 were considered statistically significant.

## 3. Results

One hundred thirty-seven adolescents participated in the study, a greater number of males (62.3%) were in the intervention, and more females (60.3%) were in the control group. Majority (70.1%) of the participants had primary school education. A higher proportion (77.9%) of the guardians also had formal education. Participants in the intervention group (85.3%) had most of their guardians having formal education training compared to the control group (70.6%). A relatively higher proportion of participants in the control group (82.4%) had majority of their guardians' classified in the low-income status as against the intervention group (63.8%) (Table 2).

Table 3 presents the knowledge level of iron between the study groups. At baseline, a greater proportion of the adolescents in the control group knew general signs of anaemia, causes of anaemia, consequences of anaemia, prevention of anaemia, iron-rich foods, iron-enhancing foods, and iron-inhibiting foods. The difference in the knowledge level of the causes of anaemia, consequences of anaemia, and iron-enhancing foods of participants in the control group and intervention group was statistically significant (*p* < 0.05). At postintervention, majority of the adolescents in the intervention group, compared to the control group, knew general signs of anaemia, causes of anaemia, consequences of anaemia, iron-rich foods, iron-enhancing foods, and iron-inhibiting foods except the prevention of anaemia. A statistically significant (*p* < 0.05) difference was observed in the knowledge level of general signs of anaemia, causes of anaemia, iron-enhancing foods, and iron-inhibiting foods of participants.

Knowledge and iron-rich food intake statuses between the study groups is shown in Table 4. At baseline, majority of adolescents in the intervention group had poor knowledge status and food intake status. However, a higher proportion of participants in the intervention group had good knowledge status and iron-rich food intake status at postintervention.

Table 5 shows knowledge and iron-rich food intake scores within the study groups. The mean knowledge scores at pre- and postintervention were statistically significant (*p* < 0.05) within both groups. The mean difference (MD) was higher in the intervention group (MD = 4.6) than in the control group (MD = 2.7). At pre- and postintervention, the difference in the mean food intake score within the control group (MD = 0.2) was slightly higher than the intervention group (MD = 0.1).

Table 6 indicates the knowledge status and iron-rich food intake score within the study groups. At baseline, participants in the control group with poor knowledge status (1.5 ± 0.8) had a higher mean food intake score compared to those with good knowledge status (1.2 ± 0.4). At postintervention, participants with good knowledge status (2.6 ± 1.3) had more mean food intake scores relative to those with poor knowledge status (1.6 ± 0.9) in the control group. The difference was statistically significant (*p* < 0.05). Participants in the intervention group with good knowledge status (2.1 ± 1.3) had a greater mean food intake score compared to those with poor knowledge status (1.8 ± 1.0). The mean difference was not statistically significant (*p* > 0.05).

Table 7 summarizes the knowledge and food intake scores before and after the intervention between the study groups. At baseline, the control group (1.1 ± 1.7) had a higher mean knowledge score than the intervention group (0.6 ± 1.0). The mean knowledge score difference was statistically significant. Also, the control group (1.4 ± 0.8) had a slightly higher mean food intake score compared to the intervention group (1.3 ± 0.8). At postintervention, the mean knowledge score was relatively greater in the intervention group (5.3 ± 1.7) than in the control group (3.9 ± 1.9). A statistically significant (*p* < 0.05) difference was observed in the mean knowledge score. No difference was observed in the mean food intake score of the groups.

## 4. Discussion

Developing nutrition interventions with the inclusion of nutrition education to improve dietary intake is indispensable in the life of adolescents. Evidence suggests that nutrition education intervention improves cognitive abilities, nutrition knowledge, and micronutrient status of children [38, 44]. The present study evaluated the impact of nutrition education on the knowledge of iron and iron-rich food intake practices of young adolescents in rural communities in Ghana. The study showed that nutrition education improved participants' knowledge level of general signs of anaemia, causes of anaemia, consequences of anaemia, iron-rich foods, iron-enhancing foods, and iron-inhibiting foods in the intervention group than the control group except for prevention of anaemia. At postintervention, majority of participants in the intervention group had good food intake status compared to the control group. The mean food intake scores between the groups were the same; however, it was relatively higher in the intervention group at postintervention.

There was an improvement in participants knowledge level of iron in both groups. It was not surprising as the same questions were used in the pre- and postintervention; the participants may have become accustomed to the questions. However, majority of participants in the intervention group increased their knowledge level of iron. A higher proportion of the participants in the intervention group compared to the control group increased their knowledge level of the general signs of anaemia, causes of anaemia, iron-enhancing foods, and iron-inhibiting foods as it showed a statistically significant difference, indicating that nutrition education was effective in enhancing the knowledge level of participants on anaemia and iron. This study adds to the others that showed that nutrition education intervention improves nutrition knowledge level of adolescents [32–34, 48, 49].

High nutrition knowledge deficit has been reported among adolescents living in some urban communities in Ghana [25]. Findings from the current study showed that majority of participants in both groups had poor knowledge status at baseline, indicating that most adolescents living in Ghana may have low nutrition knowledge. However, at postintervention, majority of participants within the intervention group had good knowledge status, and it was statistically significant. Also, participants with good knowledge status in the intervention group had a higher mean knowledge score than those in the control group. The intervention group had a higher mean difference in the knowledge score. This shows an improvement in the knowledge status of participants in the intervention group.

The present study showed no statistically significant difference in pre- and postintervention mean iron-rich food intake scores within and between the study groups. At baseline, the control group had a slightly higher mean iron-rich food intake score compared to the intervention group. At postintervention, the groups had the same mean iron-rich food intake score; however, the score was slightly higher in the intervention group. The increase in the knowledge level of participants and formal education level of the guardians in the intervention group might have contributed to the improvement in the dietary intake observed at postintervention. Guardians' having formal education are concerned about the nutrition and dietary intake of their children [26]. This finding is consistent with Chung and Fong [41] and Yahia et al. [39] studies that documented that adolescents with high nutrition knowledge improved micro- and macronutrients food intake practices.

Contrary to the baseline outcome of the iron-rich food intake status within the groups, majority of participants in the intervention group were within the good food intake status at postintervention. A statistically significant difference was observed in the iron-rich food intake status in relation to food intake scores within the study groups. This may be attributed to the fact that majority of the guardians in the study had formal education. Guardians with formal education had a positive influence on the food intake practices of their children [19, 40, 47, 50].

Despite the increase in the knowledge level and knowledge status of participants in the intervention group compared to the control group at postintervention, our study showed no statistically significant difference between participants with poor knowledge status and good knowledge status concerning their food intake score in the intervention group at postintervention. Participants in the control group showed otherwise. Although, in both groups, participants with either poor knowledge status or good knowledge status had a slight increase in the mean food intake score, this shows that an increase in nutrition knowledge may not be enough to improve food intake, as some studies have alluded to factors such as socioeconomic status, taste, and flavour influence outcomes [22, 23]. Kigaru et al. [29] found a disconnection between nutrition knowledge and food intake practices. Our study was conducted in communities in a rural district while the study by Kigaru et al. was carried out in an urban suburb in Nairobi City; the difference in the study settings and socioeconomic factors might have led to contrary outcomes. Moreso, the nutritional needs of our participants took center stage in the nutrition education intervention. A study by Addo et al. [44] on nutrition education intervention in Ghana among adolescents showed an inverse relationship between knowledge and practices. The outcome differences may be due to the duration of the intervention's implementation and possible influence of the food environment or availability. A study in Ghana also concluded that nutrition knowledge was not proportional to dietary intake [31]. The differences in the outcomes may be due to the different study designs employed, while the current study was an intervention study the others were cross-sectional studies.

### 4.1. Strength and Limitation

Nutrition education on anaemia and iron foods enlightened participants. The application of knowledge on iron-rich foods and anaemia can help to prevent and reduce the prevalence of anaemia in adolescents. Data collection on dietary intake depended on memory, and that could contribute to recall bias.

## 5. Conclusion

Nutrition education improved adolescents' knowledge of anaemia and foods related to iron. The intervention group had more participants who had a good food intake status compared to the control group at postintervention. Nutrition education should continuously be part of the education curriculum and interventions to encourage healthy eating practices among adolescents. Nutritionists and dieticians should be engaged in implementing nutrition and dietetic interventions in research. As knowledge alone seems not enough to influence healthy eating practices, we further recommend that government and nongovernmental organizations promote programmes to empower guardians economically to increase household food purchasing power.

## Figures and Tables

**Table 1:** Lesson plan for nutrition education.

Session title	Content
1. Iron	Importance and functions
	Types
	Deficiency

2. Anaemia	Causes, signs and symptoms, consequences, and prevention

3. Food sources of iron	Animal sources (specific parts of animals)
	Plant sources
	Rich, good, and poor sources

4. Foods that increase iron absorption	Micronutrient that aids iron absorption
	Foods sources
	Role of iron-enhancing foods

5. Foods that decrease iron absorption	Food inhibitory factors
	Foods sources
	Role of iron-inhibiting foods

6. Review	Relevant previous knowledge

**Table 2:** Characteristics of the participants and guardians by study groups.

Study characteristics	Total (*N*, %)	Intervention group	Control group	*X* ^2^	*p* value
Gender					
Boys	70 (51.1)	43 (62.3)	27 (39.7)	7.0	0.010
Girls	67 (48.9)	26 (37.7)	41 (60.3)		
Level of education participants					
Primary	96 (70.1)	50 (72.5)	46 (67.6)	0.4	0.579
Junior high school	41 (29.9)	19 (27.5)	22 (32.4)		
Guardians					
Formal education	106 (77.9)	58 (85.3)	48 (70.6)	4.3	0.062
No formal education	30 (22.1)	10 (14.7)	20 (29.4)		
Frequency of deworming					
Regularly	10 (7.3)	5 (7.2)	5 (7.4)	0.0	1.000
Occasionally	127 (92.7)	64 (92.8)	63 (92.6)		
Iron supplement					
Yes	114 (83.2)	56 (49.1)	58 (50.9)	0.4	0.649
No	23 (16.8)	13 (56.5)	10 (43.5)		
Frequency of iron supplement intake					
Regularly	7 (5.1)	7 (10.1)	0 (0.0)	7.7	0.006
Occasionally	130 (94.9)	62 (89.9)	68 (100)		
Guardians income status					
Low	100 (73.0)	44 (63.8)	56 (82.4)	6.0	0.020
Moderate	37 (27.0)	25 (36.2)	12 (17.6)		

Frequency (percentage); chi-square (*X*^2^); *p* value is significant at <0.05.

**Table 3:** Knowledge level of iron between the study groups at pre- and postintervention.

Characteristics	Preintervention				Postintervention			
	Control group	Intervention group	*X* ^2^	*p* value	Control group	Intervention group	*X* ^2^	*p* value
General signs of Anaemia								
Know	16 (23.5)	15 (21.7)	0.1	0.840	29 (58.0)	40 (80.0)	5.7	0.030
Do not know	52 (76.5)	54 (78.3)			21 (42.0)	10 (20.0)		
Causes of Anaemia								
Know	12 (17.6)	3 (4.3)	6.2	0.015	22 (44.0)	37 (74.0)	6.2	0.004
Do not know	56 (82.4)	66 (95.7)			28 (82.4)	13 (26.0)		
Consequences of Anaemia								
Know	13 (19.1)	5 (7.2)	4.2	0.046	29 (58.0)	37 (74.0)	2.9	0.139
Do not know	55 (80.9)	64 (92.8)			21 (42.0)	13 (26.0)		
Prevention of anaemia								
Know	12 (17.6)	9 (13.0)	0.6	0.486	43 (86.0)	41 (82.0)	0.3	0.786
Do not know	56 (82.4)	60 (87.0)			7 (14.0)	9 (18.0)		
Iron-rich foods								
Know	8 (11.8)	4 (5.8)	1.5	0.243	34 (68.0)	42 (84.0)	3.5	0.100
Do not know	60 (88.2)	65 (94.2)			16 (32.0)	8 (16.0)		
Iron-enhancing foods								
Know	17 (25.0)	8 (11.6)	4.1	0.049	30 (60.0)	43 (86.0)	8.6	0.006
Do not know	51 (75.0)	61 (88.4)			20 (40.0)	7 (14.0)		
Iron-inhibiting foods								
Know	1 (1.5)	0 (0.0)	1.0	0.496	8 (16.3)	26 (52.0)	4.0	<0.001
Do not know	67 (98.5)	69 (100)			41 (83.7)	24 (48.0)		

Frequency (percentage); chi-square (*X*^2^); *p* value is significant at <0.05.

**Table 4:** Knowledge status and knowledge scores, iron-rich food intake status, and food intake scores within the study groups.

Characteristics	Control group				Intervention group			
	*N* (%)	M ± SD	MD	*p* value	*N* (%)	M ± SD	MD	*p* value
*Baseline*								
Knowledge status								
Poor	62 (91.2)	0.7 ± 1.2	4.6	<0.001	68 (98.6)	0.5 ± 0.8	4.5	
Good	6 (8.8)	5.3 ± 0.5			1 (1.4)	5.0±		
Food intake status								
Poor	61 (89.7)	1.3 ± 0.7	1.7	<0.001	66 (95.7)	1.2 ± 0.7	1.8	<0.001
Good	7 (10.3)	3.0 ± 0.0			3 (4.3)	3.0 ± 0.0		
*Postintervention*								
Knowledge status								
Poor	27 (54.0)	2.5 ± 1.4	3.1	<0.001	12 (24.0)	2.8 ± 1.1	3.4	<0.001
Good	23 (46.0)	5.6 ± 0.7			38 (76.0)	6.1 ± 0.8		
Food intake status								
Poor	37 (74.0)	1.4 ± 0.6	2.4	<0.001	29 (58.0)	1.1 ± 0.8	2.1	<0.001
Good	13 (26.0)	3.8 ± 0.7			21 (42.0)	3.2 ± 0.4		

Frequency (percentage); mean (M) and standard deviation (SD); mean difference (MD); *p* value is significant at <0.05.

**Table 5:** Knowledge and iron-rich food intake scores within the study groups.

Variable	*N*	Control group			Intervention group		
		M ± SD	MD	*p* value	M ± SD	MD	*p* value
Knowledge score							
Preintervention	50	1.2 ± 1.7	2.7	<0.001	0.7 ± 1.0	4.6	<0.001
Postintervention	50	3.9 ± 1.9			5.3 ± 1.7		
Food intake score							
Preintervention	68	1.4 ± 0.8	0.1	0.816	1.3 ± 0.8	0.2	0.240
Postintervention	68	1.5 ± 1.4			1.5 ± 1.4		

Frequency (percentage); mean (M) and standard deviation (SD); mean difference (MD); *p* value is significant at <0.05. Paired *t*-test.

**Table 6:** Knowledge status and iron-rich food intake score within the study groups.

Study group	Knowledge status	*N*	Food intake score		
			M ± SD	MD	*p* value
Baseline					
Control	Poor	62	1.5 ± 0.8	0.3	0.160
	Good	6	1.2 ± 0.4		
Intervention	Poor	68	1.3 ± 0.8	0.8	
	Good	1	2.0±		
Postintervention					
Control	Poor	27	1.6 ± 0.9	1.0	0.004
	Good	23	2.6 ± 1.3		
Intervention	Poor	12	1.8 ± 1.0	0.2	0.555
	Good	38	2.1 ± 1.3		

Frequency (*N*); mean (M) and standard deviation (SD); mean difference (MD); *p* value is significant at <0.05.

**Table 7:** Knowledge and iron-rich food intake scores before and after the intervention between the study groups.

Study groups	Time	*N*	Knowledge score			Food intake score		
			Mean score	MD	*p* value	Mean score	MD	*p* value
Control	Baseline	68	1.1 ± 1.7	0.5	0.028	1.4 ± 0.8	0.2	0.199
Intervention		69	0.6 ± 1.0			1.3 ± 0.8		
Control	Postintervention	50	3.9 ± 1.9	1.4	<0.001	1.5 ± 1.4	0.0	0.951
Intervention		50	5.3 ± 1.7			1.5 ± 1.4		

Frequency (*N*); mean difference (MD); *p* value is significant at <0.05.

## Data Availability

Data can be accessed by writing to the Chairman, Committee on Human Research Publications and Ethics, School of Medical Sciences, Kwame Nkrumah University of Science and Technology, University Post Office, Kumasi, Ghana, or email: chrpe@knustedu.gh.
